# Acute flaccid paralysis (AFP) surveillance intensification for polio certification in Kaduna state, Nigeria: lessons learnt, 2015–2016

**DOI:** 10.1186/s12889-018-6186-y

**Published:** 2018-12-13

**Authors:** Gregory C. Umeh, Faisal Shuaib, Audu Musa, Sisay G. Tegegne, Fiona Braka, Pascal Mkanda, Richard Banda, Usman Adamu, Terna I. Nomhwange, Eyiotoyo Arenyeka, Semeeh A. Omoleke, Ticha M. Johnson, Kehinde Craig, Ibrahim Idris, Hadiza Iyal, Ishaku G. Sambo, Peter Nsubuga

**Affiliations:** 1 World Health Organization, Country Representative Office, Abuja, Nigeria; 2 National Primary Health Care Development Agency, Abuja, Nigeria; 3 Global Public Health Solutions, Atlanta, GA USA

**Keywords:** Acute flaccid paralysis, Polio certification, Kaduna state

## Abstract

**Background:**

Nigeria has made remarkable progress in its current efforts to interrupt wild poliovirus transmission despite the re-emergence of wild poliovirus in 2016. The gains made in Nigeria have been achieved through concerted efforts by governments at all levels, traditional leaders, health workers, caregivers, and development partners. The efforts have involved an elaborate plan, coordination, and effective implementation of routine immunization services, supplemental immunization activities, and acute flaccid paralysis (AFP) surveillance.

**Methods:**

We conducted the following activities to strengthen AFP surveillance in Kaduna state: a monetary reward for all AFP cases reported by health workers or community informants and verified as “true” AFP by a World Health Organization (WHO) cluster coordinator; training and sensitization of surveillance officers, clinicians, and community informants; recruitment of more personnel and expansion of the surveillance network; and the involvement of special populations (nomadic, hard-to-reach, and border communities) and caregivers in stool sample collection. The paired *t* test was used to evaluate the impact of the different initiatives implemented in Kaduna state to intensify AFP surveillance in 2016.

**Results:**

There was increased annualized non-polio AFP rate (ANPAFPR) in 21 out of 23 Local Government Areas (LGAs) of Kaduna state 6 months after implementation of different initiatives to intensify AFP surveillance. The AFP reported by the special population increased in 15 out of 23 LGAs. Statistical analyses of mean scores of ANPAFPR before and after the interventions using the paired *t* test revealed a significant difference in mean scores: mean = 19.7 (standard deviation (SD) = 16.1) per 100,000 < 15 years old in July–December 2015, compared with 38.0 (SD = 21.6) per 100,000 < 15 years old in January–June 2016 (*p* < 0.05). Likewise, analysis of silent wards using the paired *t* test showed a significant difference in mean scores: mean = 4.0 (SD = 2.1) in July–December 2015 compared with 2.4 (SD = 1.8) in January–June 2016 (*p* < 0.05).

**Conclusion:**

The different initiatives implemented in 23 LGAs of Kaduna state to intensify AFP surveillance may be responsible for the significant improvement in the AFP surveillance performance indicators in 2016.

## Background

The Global Polio Eradication Initiative (GPEI) has made tremendous gains in the last 28 years, with over 99% reduction in wild poliovirus (WPV) [1–3]. Poliomyelitis is characterized by sudden weakness or floppy paralysis of any of the limbs, most especially in children [4–8]. Acute flaccid paralysis (AFP) surveillance is the gold standard for poliomyelitis control and prevention. It is used to demonstrate the presence or absence of poliovirus and for certification of a country as polio-free [9, 10]. All cases of AFP should be investigated, and two stool samples collected from each AFP case at least 24 h apart for viral isolation in a World Health Organization (WHO)-accredited laboratory [6, 11].

Nigeria has made tremendous progress in its current efforts to interrupt WPV transmission, despite the re-emergence of WPV in 2016 [3, 12]. Nigeria has reported two WPVs from Gwoza and Jere Local Government Areas (LGAs) of Borno state 23 months after the last WPV was reported from Kano state on 24 July 2014 [12]. The greater than 90% reduction in WPV made in Nigeria has been achieved through concerted efforts by governments at all levels, traditional leaders, health workers, caregivers, and development partners [13, 14]. The efforts involved an elaborate plan, coordination, and effective implementation of routine immunization (RI) services, supplemental immunization activities (SIAs), and AFP surveillance [14–17]. The capacity of the Nigerian surveillance network to detect, report, and investigate AFP cases has been strengthened over the years through training and sensitization of surveillance officers, recruitment of more personnel, expansion of the surveillance network, and involvement of communities and caregivers in stool sample collection [14].

The greatest threats to the polio eradication program in Nigeria as shown in the 13th GPEI Independent Monitoring Board (IMB) report were suboptimal surveillance in security-compromised settlements, especially in northeastern Nigeria, and waning government commitment towards building a resilient structure against the re-emergence of poliovirus.

Nigeria still experiences late reporting and investigation of AFP cases, despite tremendous efforts made to boost AFP surveillance [18–22]. There still exist huge gaps in knowledge among health workers and caregivers of the standard case definition of AFP, and the processes of effective AFP surveillance [23]. Nigeria has wards and LGAs that are not meeting the key AFP performance indicators, such as an annualized non-polio AFP rate (ANPAFPR) of 2/100,000 of those < 15 years of age in the population and stool adequacy > 80% [22, 23]. In addition, there is little or no financial support for surveillance at the LGA level, and this has affected AFP surveillance greatly as AFP cases are investigated and transported to the laboratory late [23]. Another gap in AFP surveillance is inadequate documentation and archiving of surveillance reports, with missing records and data that could derail the certification of Nigeria as polio-free. The AFP surveillance network is skewed to health facilities in urban and semi-urban settlements, while special populations, for example nomadic, hard-to-reach, and border settlements, are neglected [19, 23]. The motivation and reward of health workers to report AFP cases is weak, leading to under-reporting [23]. Although previous studies have examined strategies in strengthening AFP surveillance in security-compromised settlements [9, 23], the current study focused on settlements not experiencing security challenges and considered other strategies.

This study evaluated all the different initiatives implemented in Kaduna state, Nigeria, in 2016 to improve AFP reporting by LGAs and to sustain gains made towards meeting standard polio certification surveillance.

## Methods

### Study design

We used descriptive and cross-sectional designs to assess the different initiatives implemented to intensify AFP surveillance in Kaduna state, Nigeria. The study described the different initiatives to intensify AFP surveillance conducted in Kaduna state, Nigeria, between January and June 2016, and also assessed their impacts when compared with AFP surveillance performance in the state in July to December 2015.

### Initiatives to intensify AFP surveillance

We implemented different initiatives to intensify AFP surveillance in Kaduna state, Nigeria, in 2016. The initiatives were monetary reward for all AFP cases reported across the board except for WHO staffers, and sensitization and re-sensitization of clinicians and community informants on AFP definition and channels of reporting. We also expanded the AFP surveillance network to include other health facilities or sites not previously prioritized for active case searches, which led to increased AFP reporting from special populations, for example nomadic, border, and hard to reach settlements, and support for documentation and archiving at various levels.

#### Monetary reward

We offered a financial reward of 1000 Naira (3 USD) for each AFP reported by health workers, community informants, voluntary community informants, teachers, LGA staff, field volunteers, and others (except for WHO staffers). The financial reward was provided by the WHO cluster coordinator who was later reimbursed after submitting proof of payments at the end of the month. There was no financial reward for rejected AFP, and payment was made after verification of the AFP as “true” AFP. Development partners were encouraged to make financial rewards during evening review meetings or at any other public function as motivation for others to report AFP. It was preferable for the Director Primary Health Care (DPHC), Local Immunization Officer (LIO), Monitoring and Evaluation Officer (M&E), or other high ranking LGA official to make the presentation to the beneficiary in the presence of partners and other stakeholders. We also implemented a quarterly reward of 10,000 Naira (34 USD) to DSNOs with highest AFP detection rate and non-polio enterovirus (NPENT) rate.

#### Sensitization and re-sensitization of clinicians and community informants on AFP definition and channels of reporting

We regularly sensitized clinicians and community informants on AFP definition and channels for reporting AFP cases. Sensitization of clinicians and community informants was conducted quarterly overall and monthly in some very busy health facilities, for example the General Hospital and the Teaching Hospital. The WHO cluster coordinator working with the hospital Matron or Medical Director was responsible for dates, timing, refreshment, and coordination of the meetings. The meeting involved discussions, lectures, exchange of contacts, distribution of IEC (Information, Education and Communication) materials, and questions and answers.

#### Expansion of AFP surveillance network to include other health facilities not previously prioritized for active case searches

We expanded the reporting sites from 150 to 180 in January 2016. The new reporting sites were carefully selected to accommodate silent and under-reporting wards. A ward is silent for AFP reporting if it fails to report at least one case of AFP in 6 months. The new reporting sites were provided with reporting tools and the focal person trained in AFP reporting and documentation.

The AFP surveillance network was expanded to nomadic, hard to reach, and border settlements. The cluster coordinator working with Ardos (Fulani leader), community, and traditional leaders identified contact persons for AFP reporting and documentation at the settlement level. The contact persons were encouraged to report immediately any cases of sudden weakness of any of the limbs to the Disease Surveillance and Notification Officer (DSNO) or the nearest reporting site. The data from nomadic, hard to reach, and border settlements were linked to the nearest reporting site.

#### Support for documentation and archiving at various levels

We provided financial and material support for documentation and archiving at a health facility, LGA, and state level. The focal persons, contact persons, and DSNOs were encouraged to adequately document and archive surveillance reports. They were also reminded of the importance of standard AFP surveillance documentation and archiving in polio certification.

### Statistical analysis

The ANPAFPR and the number of reported AFP cases by special populations were analyzed from reported AFP cases in 23 LGAs of Kaduna state 6 months before and after the initiatives to intensify AFP surveillance. Data from AFP cases reported July to December 2015 were compared with data obtained from January to June 2016 after the initiatives. We used Statistical Package for Social Sciences (SPSS) version 20 for data analysis. The paired *t* test was used to evaluate the impact of the different initiatives implemented in Kaduna state to intensify AFP surveillance in 2016.

## Results

The different initiatives implemented in Kaduna state, Nigeria, in 2016 were crucial in increasing AFP detection rates and sustaining gains made towards standard polio certification surveillance. There was a remarkable increase in funds (471%) available for training and documentation and rewards for AFP reporting (Table 1).

**Table 1:** Different initiatives implemented to intensify acute flaccid paralysis (AFP) surveillance for polio certification in Kaduna state, Nigeria, in 2016

	July–December 2015	January–June 2016
Training and sensitization	7	16
Funds for training and documentation	Naira 2,000,000 (6900 USD)	Naira 11,435,080 (39,430 USD)
Focal or reporting sites (*n*)	150	180
Reward for reporting AFP	Naira 209,000 (720 USD)	Naira 595,000 (2051 USD)
Community informants (*n*)	1203	1591

**Fig. 1:**
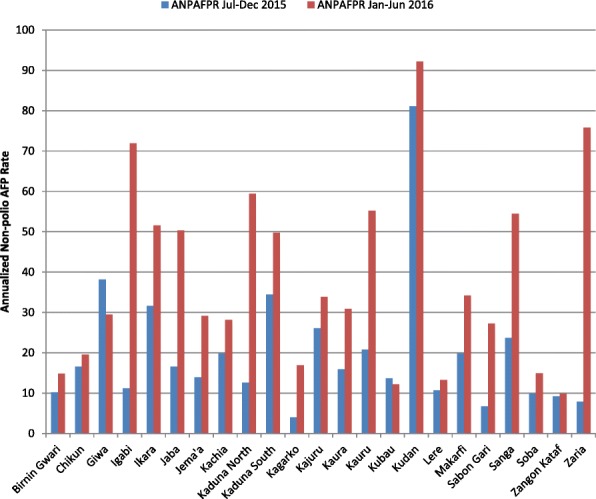
ANPAFPR annualized non-polio acute flaccid paralysis rate

There was an increase in the ANPAFPR (the same as the detection rate since Kaduna state reported no WPV, circulatory vaccine-derived poliovirus (cVDPV), or polio compatibles between January and June 2016), as shown in Fig. 1, in 21 out of 23 LGAs of Kaduna state 6 months after implementation of different initiatives to intensify AFP surveillance. The highest increase was recorded in Zaria LGA (860.0%), while Zango Kataf (7.4%) was the least improved LGA. Two LGAs, Giwa and Kubau, however, showed a decline in the ANPAFPR during the intervention period (− 22.8% and − 11.0%, respectively). The improved AFP detection was accompanied with improved surveillance report submissions, with > 80% of the LGAs achieving > 90% completeness and > 80% timeliness.

**Fig. 2:**
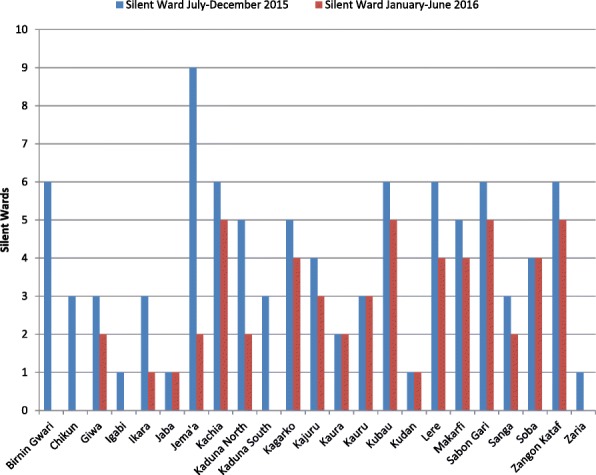
Trend of Wards not reporting Acute Flaccid Paralysis (AFP) in 23 LGAs of Kaduna State July–December 2015 and January–June 2016

There was a remarkable reduction in silent wards across the 23 LGAs of Kaduna state 6 months after implementation of various initiatives to intensify AFP surveillance (Fig. 2). A total of 55 wards out of 255 in Kaduna state were silent for AFP reporting during the intervention period compared with 92 out of 255 before the intervention.

**Fig. 3:**
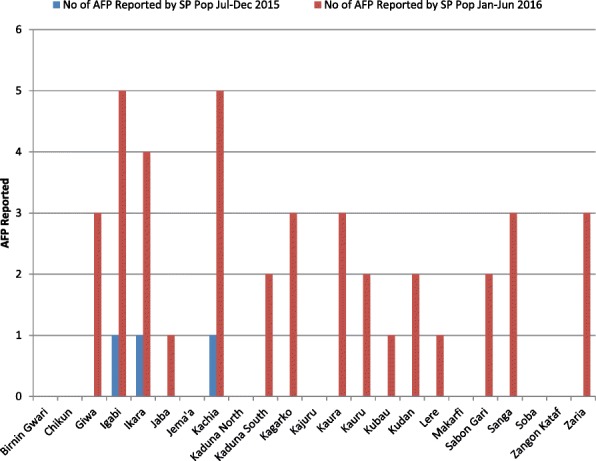
AFP acute flaccid paralysis, SP Pop special populations

The analysis of AFP reported by special populations (nomadic, hard-to-reach, and border settlements) revealed 15 LGAs reporting at least one AFP case after the initiatives were implemented compared with three LGAs before the interventions (Fig. 3). Two LGAs, Igabi and Kachia, reported five AFP cases each and accounted for 27% of the total AFP cases reported by special populations in Kaduna state.

We analyzed the mean scores of ANPAFPR before and after the interventions using paired *t* tests. The analysis showed a mean of 19.7 (standard deviation (SD) = 16.1) per 100,000 children aged < 15 years old in July to December 2015 compared with 38.0 (SD = 21.6) per 100,000 < 15 years old in January to June 2016 (*p* < 0.05). Likewise, analysis of silent wards using the paired *t* test showed a significant difference in mean scores; 4.0 (SD = 2.1) in July to December 2015 compared with 2.4 (SD = 1.8) in January to June 2016 (*p* < 0.05).

## Discussion

We found an improved annualized non-polio AFP rate (ANPAFPR) in 21 LGAs of Kaduna state, Nigeria, in 2016, likely due to the implementation of different initiatives to intensify AFP surveillance. This improvement is largely due to increased AFP reporting by LGAs, a reduction in silent and under-reporting LGAs, an increase in the percentage of LGAs meeting the key AFP performance indicators, and increased numbers of AFP cases reported by special populations. The annualized non-polio AFP rate equals the detection rate since Kaduna state reported no WPV, cVDPV, or polio compatibles between January and June 2016. The improved AFP detection was accompanied by improved surveillance report submissions, with > 80% of the LGAs achieving > 90% completeness and > 80% timeliness.

The Zaria LGA recorded the highest improvement in the annualized non-polio AFP rate, while Giwa and Kubau LGAs declined in performance. The 23 LGAs in Kaduna state met the minimum annualized non-polio AFP rate (2/100,000 < 15 years old) and stool adequacy of > 80%. Two LGAs, Igabi and Kachia, reported more AFP cases than other LGAs from their special populations.

Training and sensitization to AFP surveillance was intensified across the 23 LGAs of Kaduna state. WHO, working with the state Ministry of Health and the state primary health care agency, organized training for DSNOs, assistants DSNOs, surveillance focal persons, clinicians, and community informants. The interactions provided by the training increased the capacity of trainees and made the surveillance network more robust and sensitive.

There was increased funding for AFP surveillance documentation, which greatly assisted the state in meeting the required standards of documentation for polio certification. The funds were utilized for procurement of shelves, display boards, stationery, and archiving of surveillance reports.

The expansion of the AFP surveillance network greatly improved AFP detection and reporting. There was a 20% increase in reporting of focal sites, which are health facilities that are routinely prioritized for active case searches for AFP and other epidemic-prone diseases. All General and Teaching Hospitals and some selected Primary Health Centers, based on client flow, the population of the settlement, and geography, were included in the AFP surveillance network. The engagement of additional community informants was another boost to the sensitivity of the AFP surveillance network. The community informants included nomadic contact persons, patent medicine vendors, traditional healers, bone-setters, and community leaders.

The inclusion of financial reward for all reported AFP cases greatly encouraged DSNOs, surveillance focal persons, clinicians, and community informants to detect and report AFP. There was a remarkable increase in AFP reporting after the introduction of the financial reward, which was further strengthened by the prompt redemption of 1000 Naira (3 USD) once the AFP was verified as “true” AFP.

Our findings are consistent with a previous study by Hamisu et al. in security-compromised northeastern states of Nigeria which showed that regular training and sensitization of health workers, community informants, caregivers, and expansion of the AFP surveillance network improved AFP surveillance indicators in security-compromised settlements [23]. Furthermore, our study also showed that other interventions worked. The implementation of a monetary reward for all reported AFP cases, engagement of contact persons in special populations (i.e., nomadic, hard-to-reach, and border settlements), and material and financial support for documentation and archiving may have contributed to the improvement in AFP performance indicators.

Our findings may be relevant in strengthening AFP surveillance systems in LGAs or states with suboptimal AFP performance indicators so that the march to Nigerian certification as polio-free is realized. It may also be useful to public health physicians, epidemiologists, and clinicians in the control and prevention of emerging and other priority diseases targeted for eradication or elimination. Other countries that are yet to interrupt poliovirus transmission, especially Pakistan and Afghanistan, may benefit from our findings.

We recognize limitations in our work. Our greatest limitation was suboptimal surveillance activities in the security-compromised settlements of Birnin Gwari, Chikun, Giwa, Igabi, and Kachia LGAs of Kaduna state. These security-compromised settlements were silent for AFP reporting, have no community informants, lack functional routine immunization structure, and were largely inaccessible during SIA campaigns. These settlements were not prioritized for AFP surveillance, owing mainly to insecurity and the fact that supervisors cannot access them for active searching for AFP cases. The re-emergence of Nigeria as a polio-endemic country, following the reporting of two WPVs in Gwoza and Jere LGAs of Borno state, has shown the need for a resilient structure against the re-emergence of poliovirus [12]. However, we believe that intensifying current initiatives, implementing the recommendations of the 13th GPEI IMB report, improving AFP surveillance in security-compromised settlements, and firming up waning government commitment to polio eradication will guarantee a resilient structure against the re-emergence of poliovirus in Nigeria [12]. The sustainability of monetary rewards for all AFP reports is a limitation of this study, but ownership of the program by the government and the availability of a budget for surveillance at the state and LGA levels will ensure the program is sustained. Our study cannot infer causality, and the improvement in AFP surveillance indicators documented in Kaduna state in 2016 may be due to other factors, such as contributions of ministries of education, agriculture, and budgets not considered by the authors. However, we strongly believe the initiatives we implemented have greatly contributed to the success recorded in AFP surveillance.

## Conclusions

The different initiatives implemented in 23 LGAs of Kaduna state to intensify AFP surveillance may be responsible for the significant improvement in the AFP surveillance performance indicators in 2016. We recommend that our interventions should be replicated in other states in Nigeria and other countries in the world with suboptimal AFP surveillance. The initiatives should be sustained by government and development partners, and polio structure should be utilized to control and prevent epidemic-prone diseases, both now and after certification of Nigeria as polio-free.

## Abbreviations

AFP: Acute flaccid paralysis
ANPAFPR: Annualized non-polio acute flaccid paralysis rate
cVDPV: Circulatory vaccine-derived poliovirus
DPHC: Director of Primary Health Care
DSNO: Disease Surveillance and Notification Officer
GPEI: Global Polio Eradication Initiative
IMB: Independent Monitoring Board
LGA: Local Government Area
LIO: Local Immunization Officer
NPENT: Non-polio enterovirus
RI: Routine immunization
SD: Standard deviation
SIA: Supplemental immunization activity
WHO: World Health Organization
WPV: Wild poliovirus
